# Loss mitigation in plasmonic solar cells: aluminium nanoparticles for broadband photocurrent enhancements in GaAs photodiodes

**DOI:** 10.1038/srep02874

**Published:** 2013-10-07

**Authors:** N. P. Hylton, X. F. Li, V. Giannini, K. -H. Lee, N. J. Ekins-Daukes, J. Loo, D. Vercruysse, P. Van Dorpe, H. Sodabanlu, M. Sugiyama, S. A. Maier

**Affiliations:** 1 Department of Physics, Imperial College London, London SW7 2AZ, United Kingdom; 2 Institute of Modern Optical Technologies, Soochow University, Suzhou, Jiangsu 215006, P. R. China; 3 IMEC, Kapeldreef 75, 3001 Leuven, Belgium; 4 Department of Physics and Astronomy, Katholieke Universiteit Leuven, Celestijnenlaan 200, 3000 Leuven, Belgium; 5 Institute of Engineering Innovation, School of Engineering, University of Tokyo, Tokyo 113-8656, Japan

## Abstract

We illustrate the important trade-off between far-field scattering effects, which have the potential to provide increased optical path length over broad bands, and parasitic absorption due to the excitation of localized surface plasmon resonances in metal nanoparticle arrays. Via detailed comparison of photocurrent enhancements given by Au, Ag and Al nanostructures on thin-film GaAs devices we reveal that parasitic losses can be mitigated through a careful choice of scattering medium. Absorption at the plasmon resonance in Au and Ag structures occurs in the visible spectrum, impairing device performance. In contrast, exploiting Al nanoparticle arrays results in a blue shift of the resonance, enabling the first demonstration of truly broadband plasmon enhanced photocurrent and a 22% integrated efficiency enhancement.

The interplay between scattering and absorption in nanoplasmonic systems has recently been the subject of intense research efforts^1^,^2^,^3^,^4^. Indeed it has been shown that carefully designed metallic nanostructures can be tuned to provide a desired optical response^1^,^2^,^3^,^4^. This delicate balance of plasmonic absorption interactions versus far-field scattering properties can have implications in a number of fields, of which solar energy is a prime example^3^,^4^,^5^,^6^. Both near-field confinement as well as far-field scattering and light trapping effects have the potential to deliver absorption enhancements in solar cells^5^,^6^,^7^,^8^, however parasitic absorption in metal nanoparticles remains a key problem yet to be overcome. This is an issue of particular importance since large-scale solar cell deployment requires efficient use of materials and there is therefore a need to move towards thin-film solar cells without compromising energy conversion efficiency.

Attempts at tuning the balance between absorption and scattering have to date focused on altering the shape and dimensions of noble metal nanostructures^1^,^2^. However, in the field of solar energy where mass production is an economical necessity it is prudent to employ simple, easily producible structural parameters. We have therefore turned our attention to the use of alternative metals to redress the trade-off between scattering and absorption. In particular we perform a comparison of nanoparticles made from Au, Ag and Al. Shown in Figure 1(a) are contour plots of scattering and absorption cross-section efficiency (defined as the ratio of the scattering/absorption cross-section and the geometrical cross-section) resulting from Mie calculations of spherical Au, Ag and Al nanoparticles, displayed as a function of wavelength and nanoparticle radius. The contour plots clearly show that drastic differences in both scattering and absorption can be achieved for the different metals, even for simple nanoparticle geometries. Al in particular provides significant scattering with minimal absorption over much of the visible spectrum. We note also that Ag particles of radius >70 nm actually exhibit the lowest absorption cross-section efficiency, however these dimensions do not coincide with those required for maximum scattering. In fact only in the case of Al do the conditions for maximum scattering also result in low absorption, making it an attractive candidate for solar energy applications. We also note that Al has recently been gathering interest as a plasmonic material since it presents the opportunity to shift plasmonic resonances away from visible frequencies and into the ultraviolet^9^,^10^.

Indeed a recent article presented the results of finite difference time domain calculations demonstrating the potential of Al nanoparticles to provide broadband absorption enhancements in Si films^11^. Another report proposed the use of Al as a suitable material for plasmonic enhancements in organic devices, however this work was limited to purely optical considerations^12^. The authors observed increased extinction when Al nanoparticles were embedded in organic layers, but this includes absorption in the particles and no data were presented regarding photocurrent measurements^12^. We note however, that the existence of parasitic absorption highlights the fact that overall absorption/extinction enhancements do not necessarily translate into efficiency gains, thereby underlining the need for photocurrent measurements, rather than purely optical characterization. In this paper we elucidate a mechanism involving scattering by metal nanoparticles that can enhance the spectrally integrated external quantum efficiency (EQE) by more than 20% and we show for the first time that losses at short wavelengths can be avoided even when using nanoparticles on the front surface. We present the results of an experimental comparison between GaAs photodiodes incorporating Au, Ag and Al periodic nanoparticle arrays and a reference device. These data are supported by comprehensive electromagnetic and electronic simulations performed using a simulation tool we recently developed^13^,^14^. We demonstrate that the parasitic losses commonly observed at short wavelengths are intimately linked with the properties of the metal and/or the excitation of localized surface plasmons. The losses therefore depend strongly on the choice of metal and structural parameters used and can in fact be avoided when using Al nanoparticles, resulting in broadband photocurrent enhancements. Furthermore, we highlight the delicate balance between broadband optical path length increases arising from scattering, and parasitic absorption due to the coupling of incident light into nanoparticle plasmon resonances.

Considerable recent effort has been placed into nanoscale light trapping and plasmon-enhanced solar cells since arrays of sub-wavelength metallic nanoparticles can be used to scatter incident light into the active region. If the incident light is scattered through a sufficiently high angle then the optical path length in the device can be significantly increased compared to a flat cell. This approach has led to some improvement in the long-wavelength (λ > 550 nm) photocurrent response of both GaAs and Si solar cells when Ag or Au nanoparticles are fabricated on the surface of the cell, although these types of devices typically suffer from reduced photocurrent at shorter wavelengths^15^,^16^,^17^,^18^. Recently, the attribution of such long-wavelength increases in photocurrent to plasmonic scattering effects has been verified through detailed optical and electronic simulations^13^,^14^. Light scattering in this manner opens up the potential for the fabrication of devices that are optically thick but physically thin, thus reducing the amount of raw material used and hence the cost of the solar cell, without compromising its efficiency. To realize this goal however, the trade-off between enhancements brought about through scattering and the characteristic deficit observed at shorter wavelengths must be fully understood and controlled. Some reports have suggested that the increase in EQE on the red side of the spectrum is the result of constructive interference between incident and scattered waves, while in the short wavelength regime destructive interference has been blamed for the drop in EQE relative to bare cells^19^,^20^. More recently however, parasitic absorption in the metal nanoparticles has also been proposed as a culprit for reduced EQE, since short wavelength photons couple strongly with resonant nanoparticle plasmon modes rather than scattering into the semiconductor material beneath^21^. Nevertheless it is clear that the problem of loss at short wavelengths is inherent to the use of Au and Ag nanoparticle arrays positioned on the front surface of the photovoltaic cell. Alternative methods that have therefore been proposed to alleviate parasitic losses include the use of dielectric^22^,^23^,^24^,^25^,^26^ rather than metallic nanostructures, or rear surface textured plasmonic reflectors^27^,^28^,^29^,^30^, both of which have yielded some positive results. Despite this progress we believe that appropriate metallic structures on the front surface of the photovoltaic cell may still prove useful in providing optical path length enhancements. For this approach to be a viable addition to conventional anti-reflection coatings however, the problem of parasitic absorption losses must be mitigated. The choice of metal employed for the scattering nanoparticle arrays may therefore prove crucial in optimizing photocurrent enhancements and so a good understanding of the optical response of different metallic nanoparticles is required. To this end we devised a comparison between the photocurrent response of thin-film GaAs photodiodes with periodic arrays of Au, Ag and Al nanoparticles patterned on the front surface. A schematic diagram of the complete photodiode structure is presented in Figure 1(b), and an example scanning electron microscope (SEM) image of one of the Al nanoparticle arrays is shown in Figure 1(c), demonstrating the high nanoparticle uniformity and regular spacing. The nanoparticle diameter, height and pitch are 100, 50 and 200 nm respectively.

## Results

Plotted in Figure 2(a) are EQE spectra measured for photodiodes with nanoparticle arrays of each different metal fabricated on the top surface and a reference. The reference sample exhibits typical photocurrent response across the visible and near-infra red parts of the spectrum up to the GaAs band edge. The EQE rises from the GaAs band gap close to 880 nm, peaks at around 31% and drops off once more at short wavelengths where carriers are lost to surface recombination. Note that the relatively low peak value of the EQE arises from the short carrier diffusion length. With the application of a Au nanocylinder array of 200 nm pitch the form of the EQE spectrum is distinctly modified however (blue curve in Figure 2(a)). At wavelengths longer than 600 nm we observe increased EQE with respect to the reference, which we ascribe to reduced surface reflection and increased optical path length from forward scattering of incident photons into the device. In contrast the EQE is significantly reduced at shorter wavelengths as a result of absorption due to interband transitions and resonant plasmon modes in the Au particles. Next we turn our attention to an equivalent array of Ag nanoparticles (red curve); in this case the losses at short wavelengths remain present but are reduced compared to the Au nanoparticles, consistent with differences in the complex dielectric functions of the two metals due to the fact that the interband transitions are not present. Note additionally that this means a greater portion of the spectrum exhibits photocurrent enhancement from far-field scattering effects. Also plotted in Figure 2(a) are EQE spectra of photodiodes with Al nanoparticle arrays of equivalent (200 nm, green curve) and larger (400 nm, light blue curve) pitches. Considering first the 200 nm pitch array, we observe that enhancement from scattering occurs over the majority of the spectral region of interest, i.e. when λ > 500 nm, and that the losses are minimal at shorter wavelengths. Crucially though, these losses can be avoided entirely by increasing the pitch of the nanoparticle array to 400 nm, i.e. reducing the metal coverage; here we observe spectrally broad enhancement in the EQE from 400–900 nm. Indeed this larger pitch array of Al nanoparticles outperformed all other arrays fabricated from either Au or Ag. This we attribute to the fact that the plasma frequency of Al occurs at a higher energy than Au and Ag, i.e. in the spectral region studied here Al acts as a very good Drude metal with small penetration of the electric field into the nanoparticles and therefore low optical absorption due to the nanoparticles.

Corresponding simulations of the optical and electronic response of GaAs photodiodes with Au, Ag and Al nanocylinders were performed and the resulting calculated EQE spectra are plotted in Figure 2(b). For completeness we have extended the simulations to also include Au and Ag nanoparticle arrays of 400 nm pitch, the results of which can be found in the supplementary information. The simulations were carried out using a 3D finite element method, where the electromagnetic problem is directly linked to carrier transport calculations^13^,^14^. In other words we calculate the solar cell performance by concurrently solving the Maxwell equations of a plane wave incident on the solar cell and the electronic response (carrier transport calculations) generated by the scattered electric field^13^,^14^. Note that we have not included any nanometric oxidation of the nanoparticles in any of the simulations. When comparing these EQE spectra to that of a simulated reference device we observe that the effect of adding Au, Ag and Al nanocylinders on the form of the spectra follows the same trend as observed experimentally. Namely, we again note that despite providing scattering enhancements at long wavelengths the Au nanoparticles exhibit significant detrimental absorption below 550 nm. Moreover, when we switch first to Ag, then Al, the losses can be reduced and ultimately eliminated. However in the simulations the Al array of 200 nm pitch outperforms that of 400 nm. The small discrepancies between simulation and experiment are due to the geometrically perfect nanoparticles and the dielectric functions used in the simulations. In these calculations we used dielectric constants measured and tabulated by Palik^31^ that can present some difference from the true dielectric functions. Additionally, we solved the problem for geometrically perfect cylinders, however small imperfections in the shape of the fabricated nanoparticles can give rise to important differences in the plasmonic response thus accounting for the difference between simulated and experimental results^3^,^4^. Nevertheless we want to stress that, as shown in Figure 2, the physical behaviour and magnitudes obtained in the simulations match very well with those of the experiments. Furthermore, the discrepancy highlights the intricate balance between forward scattering and nanoparticle absorption, which is of prime importance for optimal EQE. Comparing the simulations and the experiments we can gain an important indication of the ideal nanostructure parameters. For example, based on our experiments and simulations and for our chosen cylinder dimensions we expect that the optimum pitch should lie between 200 and 400 nm. A more detailed optimization is possible and would require investigations into the effect of varying nanoparticle radius and height, as well as the pitch, but we want to stress that this is outside the scope of this work as we are focusing on the choice of metal as a scattering medium.

Our results are also supported by a recent report of the results of finite difference time domain calculations of optical absorption in Si layers with spherical metal nanoparticles on the surface^11^. The reason for this change in the observed losses is that the plasma frequency of the metals shifts from the visible part of the spectrum towards the ultraviolet as we move from Au to Ag and into the deep-UV for Al^32^. Therefore the detrimental parasitic absorption in the metal particles and destructive interference effects also shift to shorter wavelengths and, in the case of Al, out of the relevant spectral window^31^. Furthermore, this also opens up new possibilities for the use of other scattering metals with high plasma frequencies.

To further demonstrate the great potential posed by Al nanoparticle arrays we present a bar chart in Figure 2(c) showing the fractional efficiency enhancement provided by each of the nanoparticle arrays, spectrally integrated from the experimental data. It is clear from the chart that Au and to a lesser extent Ag nanoparticles lead to an overall reduction in the integrated EQE compared to the reference diode. In contrast the Al arrays provide around a 6% and 22% increase for the arrays of 200 nm and 400 nm pitch respectively. This finding has significant implications for the enhancement of full solar cell devices; by combining the scattering properties of our Al nanoparticle arrays with conventional antireflection coatings, significant steps could be made towards thinner devices while maintaining the high power conversion efficiencies associated with GaAs cells.

To emphasize the differences between the metals considered here, we next consider the absorption in the nanoparticles themselves. Plotted in Figure 3(a) are absorbance spectra calculated for arrays of Au (blue line), Ag (red) and Al (green) nanoparticles, where arrows indicate the plasmon resonances (which are damped by the high index substrate). We observe strong, broad absorbance in the Au nanoparticles due to interband transitions in the metal overlapping with two plasmon resonances. The absorbance of the Ag nanoparticle array is also significant at short wavelengths and exhibits two plasmon resonances, but without the contribution from interband transitions. Our simulations indicate that, as reported by Beck *et al.*^21^, these two resonances can be attributed to the excitation of different plasmonic modes localized either at the top or the bottom of the nanoparticles (see supplementary information). In contrast, the Al nanoparticle array exhibits low absorbance (<0.1) over much of the relevant spectral region. In fact the Al absorbance only peaks at the localized surface plasmon resonance in the UV and remains at a value significantly lower than that of the other two metals (here we only observe the resonance at the bottom of the particle and expect the resonance at the top to be at shorter wavelengths). This very low absorbance we attribute to the high dielectric constant of the Al resulting in a short penetration depth of the electric field into the particles. i.e. the short field penetration means that the parasitic absorption is low in Al even at the plasmon resonance at 380 nm (see Figure 3), while beneficial scattering remains at longer wavelengths. This is especially true since it has been suggested^19^,^20^ that constructive interference occurs between incident and scattered waves at wavelengths longer than the plasmon resonance. As an aside it should be noted that at long wavelengths (λ > 700 nm) optical absorption in the Al particles is slightly increased compared to Au or Ag due to a weak interband transition close to 800 nm. Overall though, absorption losses in Al particles are significantly lower than for Au or Ag.

Next we turn our attention to the anti-reflection properties of metal nanoparticle arrays. Shown in Figure 3(b) are simulated reflectance spectra from each of the photodiodes. The reference device (black curve) exhibits a reflectance of around 20% across much of the visible spectrum but this value rises in the near UV and at longer wavelengths near the band edge of the underlying GaAs material. For all three metals considered here a reduction in reflectance was observed at wavelengths from 700–900 nm. In the visible and near UV parts of the spectrum however, peaks in the reflectance can be observed that coincide with the excitation of localised plasmon resonances. The reflectance maxima are characteristic of destructive interference effects that have been reported elsewhere^19^,^20^ and occur slightly blue shifted with respect to the resonance peaks. Their presence means that for the cases of both Au and Ag there are reflectance peaks in the crucial spectral range of 400–700 nm while the reflectance from the Al device remains below 10% in this range and rises to around 25% at 360 nm. The peaks in the reflectance spectra for the Au and Ag are centred at wavelengths of 600 and 500 nm and reach values of 35 and 45% respectively. These represent significant losses but we note that the peak values of absorbance in the nanoparticles are somewhat higher (up to 65% for Ag) and the spectral width is greater (particularly in the case of Au), emphasizing the crucial role played by parasitic absorption in these types of devices.

Finally, in Figure 3(c) we plot colour maps of the absorption taken as a vertical slice through the centre of the devices at λ = 500 nm. In the case of Au the absorption in the nanoparticle is high and we see modest absorption in the GaAs beneath the particle from the scattered field. The absorption in the GaAs is increased in the case of Ag and we observe a modification to the absorption profile in the form of distinct maxima and minima, arising from light coupling into modes within the absorbing layer. However, absorption in the particle itself remains high, to the detriment of the solar cell performance. Finally, the Al array exhibits strong delocalized optical absorption in the GaAs, but crucially the absorption in the particle remains low and the small field penetration into the metal can clearly be seen.

## Discussion

To conclude, a comparison of the photocurrent response of GaAs photodiodes with Au, Ag and Al nanocylinder arrays was carried out to quantify the potential for efficiency enhancements in III-V photovoltaic cells. By comparing EQE measurements to that of a reference diode we demonstrated that although all of the nanoparticle arrays provided scattering enhancements at long wavelengths, the losses suffered by the Au and Ag arrays at short wavelengths dominate, rendering these arrays impractical. In the case of Al nanoparticles on the other hand, parasitic losses were minimized and scattering effects dominated, leading to broadband enhancement in the EQE. We showed that using such Al nanoparticle arrays we were able to achieve an increase of 22% in the spectrally integrated EQE. This type of broadband enhancement opens up the possibility to move towards producing solar cells with thin-film absorbers, without compromising power conversion efficiencies, thus reducing material consumption. Finally we also provide the results of simulations showing device reflectance as well as the absorption in critical regions of the photodiode. These results highlight the crucial role played by the scattering particle and underline the need to carefully choose an appropriate material, such as Al.

## Methods

### Growth and fabrication

**Figure 1:**
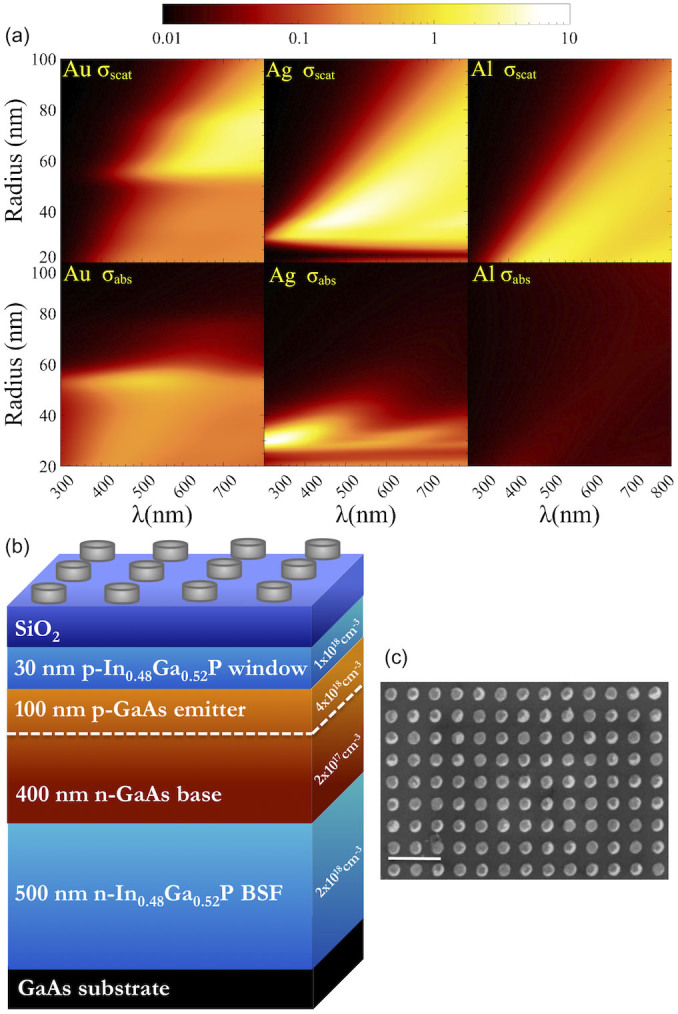
Light scattering and absorption using metal nanoparticle arrays. (a) Contour plots showing scattering and absorption cross-section efficiency calculated using Mie theory for spheres of Au, Ag and Al (all plots use an identical logarithmic scale bar). (b) a schematic diagram of the p-n junction photodiodes with an array of nanocylinders on the front surface (the composition, thickness and doping density of each layer is indicated on the diagram), and (c) SEM image of a periodic array of Al nanocylinders fabricated by electron beam lithography (scale bar is 500 nm).

**Figure 2:**
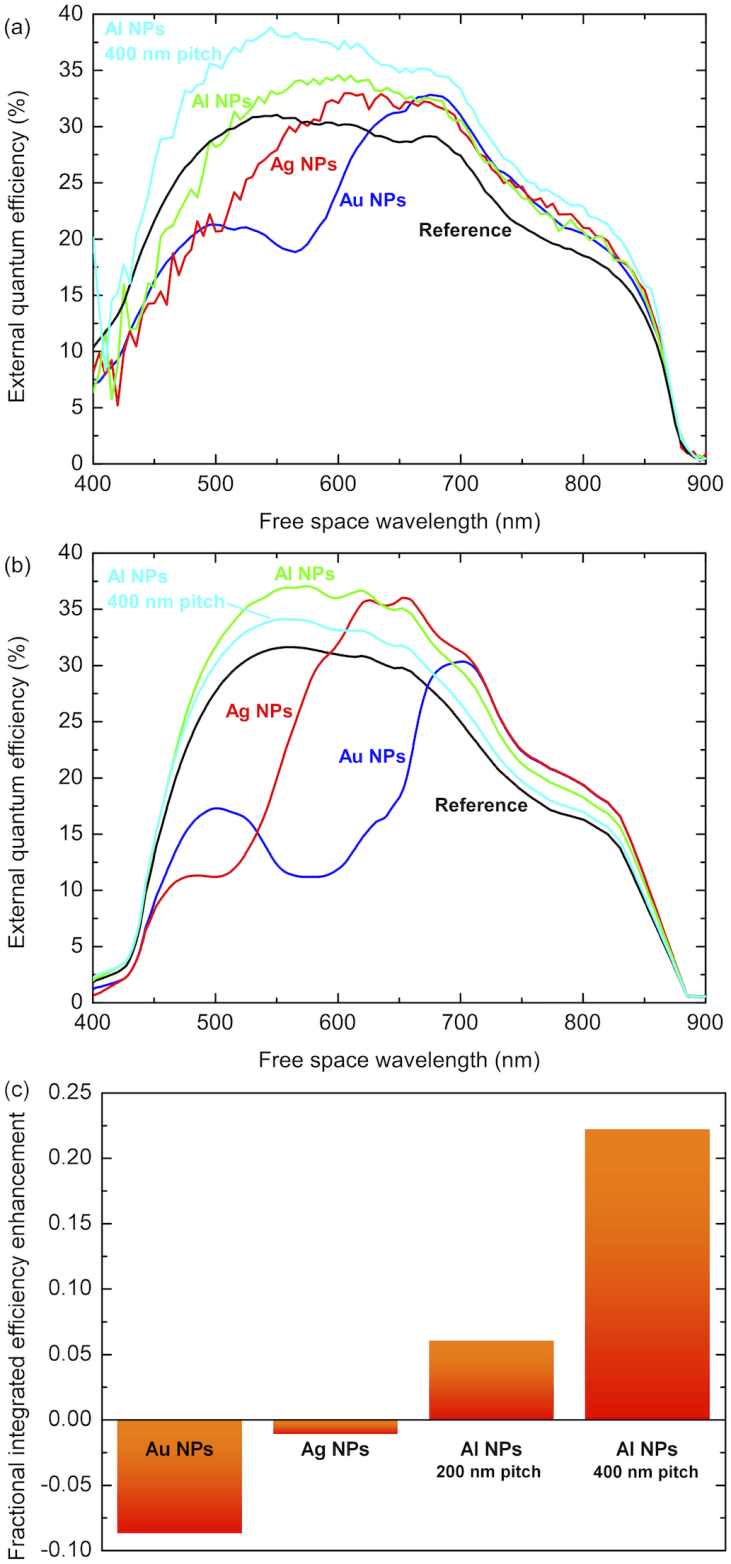
Experimental and calculated photocurrent spectra and enhancement ratios. (a) Experimentally observed and (b) numerically calculated EQE spectra plotted as a function of wavelength for a reference photodiode (black line) and devices with periodic Au (blue), Ag (red) and Al (green) nanoparticle arrays of 200 nm pitch and an Al array of 400 nm pitch (light blue). (c) Integrated EQE enhancement provided by each of the nanoparticle arrays plotted with respect to the reference device, taken from the experimental data.

**Figure 3:**
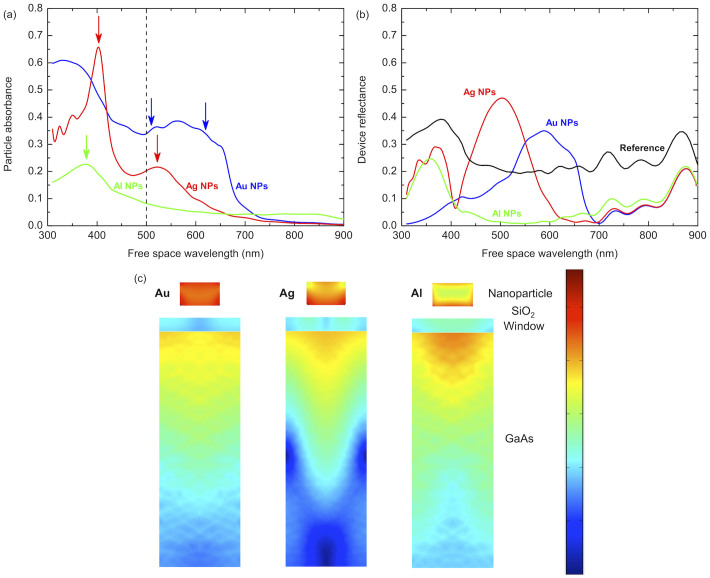
Simulated optical response of the nanoparticles and device active region. (a) Absorbance of the metallic nanoparticles plotted as a function of wavelength for Au (blue), Ag (red) and Al (green) and (b) the simulated reflectance spectra for those devices and the reference (black). (c) Vertical slice colour maps through the device showing the absorption at λ = 500 nm in the nanoparticles, window layer and p-n junction regions for Au, Ag and Al arrays (all colour maps use an identical logarithmic colour scale).

GaAs p-n junction photodiodes of 500 nm thickness were grown on GaAs substrates by metal-organic vapour phase epitaxy, employing In_0.48_Ga_0.52_P for both the window and back surface field layers (doping concentrations for each layer are indicated in Figure 1(b)). Following the growth stage the samples were processed into isolated, ring-shaped mesa diodes with an optical window of diameter 600 μm. Finally a 25 nm spacer layer of SiO_2_ was deposited on the surface of the samples prior to fabrication of Au, Ag and Al nanoparticle arrays by electron beam lithography. Periodic arrays of cylindrical nanoparticles with a diameter of 100 nm, height of 50 nm and pitch of 200 were fabricated in each metal type plus an additional array of Al nanoparticles with 400 nm pitch. Reference mesa diodes underwent identical processing steps up to and including the SiO_2_ deposition. For the purposes of this experiment the photodiode samples were fabricated with the intention of achieving a short minority carrier diffusion length. This means that only carriers that are photo-generated in the depletion region can be captured and contribute to photocurrent. In a measurement of quantum efficiency we therefore only sample a thin layer of absorbing material, which is an ideal probe to monitor the effects of the scattering nanoparticle arrays.

### Measurements

Photocurrent measurements were carried out using a Halogen lamp connected to a Bentham Instruments monochromator to excite the devices. The monochromator output was coupled to a multimode optical fibre, delivered to a custom built microscope system and focused to a spot of diameter ~90 μm. Photocurrent was recorded using standard lock-in techniques and subsequently converted into EQE via measurements with a calibrated detector.

## Author Contributions

N.P.H., X.L., V.G., K.H.L., N.J.E.D. and S.A.M. conceived the study and discussed the data. H.S. and M.S. grew the photodiodes and J.L., D.V. and P.V.D. carried out the nanoparticle fabrication and scanning electron microscopy. N.P.H. carried out the experimental characterization and N.P.H., X.L. and V.G. performed the simulations. The manuscript was written by N.P.H., and reviewed and approved by all other authors.

## Supplementary Material

Supplementary Information Loss mitigation in plasmonic solar cells: aluminium nanoparticles for broadband photocurrent enhancements in GaAs photodiodes - Supplementary information
