# Supplementary material for: Loss mitigation in plasmonic solar cells: aluminium nanoparticles for broadband photocurrent enhancements in GaAs photodiodes
Source: Sci Rep. 2013 Oct 7;3:2874. doi: 10.1038/srep02874 (PMC3791440; doi:10.1038/srep02874)
Supplement: Supplementary Information — Loss mitigation in plasmonic solar cells: aluminium nanoparticles for broadband photocurrent enhancements in GaAs photodiodes - Supplementary information [file srep02874-s1.pdf]

Loss mitigation in plasmonic solar cells: aluminium nanoparticles for broadband photocurrent enhancements in GaAs photodiodes – Supplementary information

N.P. Hylton[1], X.F. Li[2], V. Giannini[1], K-H. Lee[1], N.J. Ekins-Daukes[1], J. Loo[3], D. Vercruysse[3], P. Van Dorpe[3,4], H. Sodabanlu[5], M. Sugiyama[5], and S.A. Maier[1]

[1]Department of Physics, Imperial College London, London SW7 2AZ, United Kingdom, [2]Institute of Modern Optical Technologies, Soochow University, Suzhou, Jiangsu 215006, PR China, [3]IMEC, Kapeldreef 75, 3001 Leuven, Belgium, [4]Department of Physics and Astronomy, Katholieke Universiteit Leuven, Celestijnenlaan 200, 3000 Leuven, Belgium, [5]Institute of Engineering Innovation, School of Engineering, University of Tokyo, Tokyo 113-8656, Japan.

Further external quantum efficiency simulations

Plotted in Supplementary Figure S1 are simulated EQE spectra of the reference device and photodiodes with arrays of Au, Ag and Al nanoparticles of 400 nm pitch. We note that the overall trend (with respect to the reference) of the spectra for Au, Ag and Al nanoparticles is broadly similar to that for the arrays with a pitch of 200 nm. A reduction in the EQE spectra of the diodes with Au arrays can be observed at short wavelengths and these losses are reduced for the case of Ag and totally mitigated for the case of Al nanoparticles. Compared with the devices bearing nanoparticle arrays of 200 nm pitch however, the magnitude of the losses exhibited at short wavelengths by the Au and Ag arrays is reduced. This we attribute to the reduced areal coverage of the metal particles on the surface of the device.

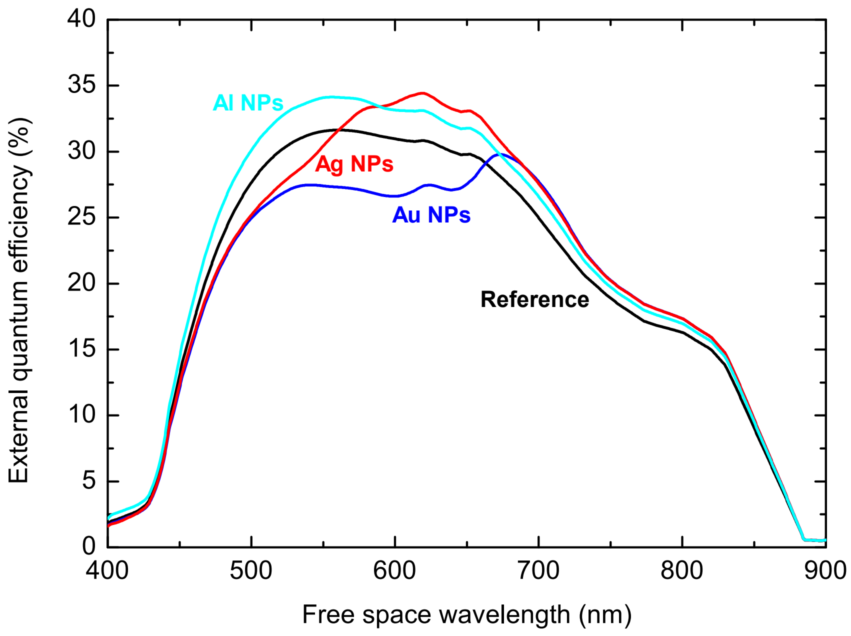

Supplementary Figure S1 Calculated external quantum efficiency spectra Numerically calculated EQE spectra plotted as a function of wavelength for a reference photodiode (black line) and devices with periodic Au (blue), Ag (red) and Al (light blue) nanoparticle arrays of 400 nm pitch.

Analysis of resonant nanoparticle plasmon modes

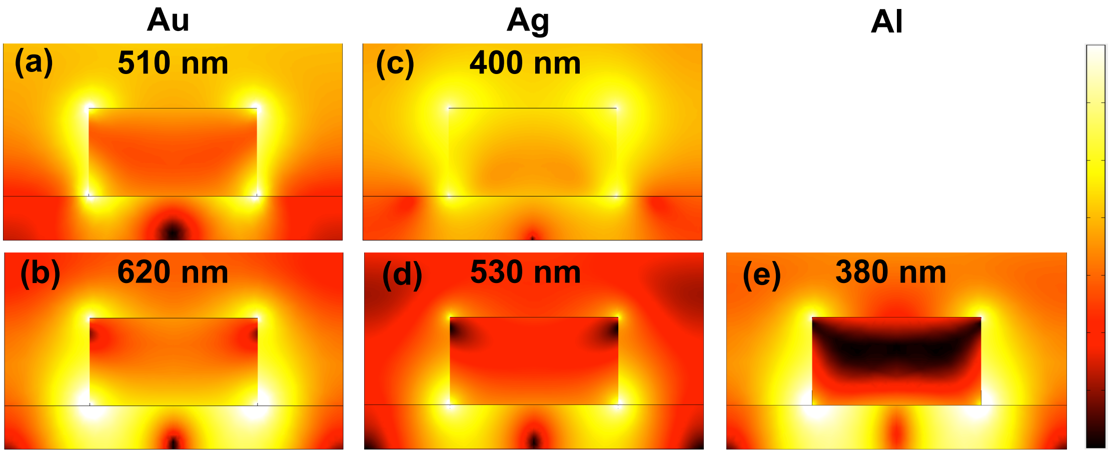

Supplementary Figure S2 Analysis of localised surface plasmon resonances Colour maps of the metal nanoparticles taken in vertical cross-section and showing the electric field intensity distribution on a logarithmic scale at the wavelengths of the plasmon resonances. Shown here are the Au particle at wavelengths of 510 nm (a) and 620 nm (b), the Ag particle at wavelengths of 400 nm (c) and 530 nm (d) and the Al nanoparticle at a wavelength of 380 nm (e).

Shown in Supplementary Figure S2 are a series of colour maps plotting the calculated electric field intensity through a vertical cross-section of the nanoparticles and the SiO_2 spacer layer on the top surface of the photodiode. The field distributions are plotted for each of the plasmon resonances indicated by arrows in Figure 3(a) of the main text. For both the Au and Ag nanoparticles we observe two localized surface plasmon resonances of different character. The short wavelength resonances at 510 nm for Au and 400 nm for Ag (Supplementary Figure S2 (a) and (c)) exhibit significant field localization at the top of the nanoparticle, i.e. at the interface with air. In contrast, the resonances at longer wavelength (620 nm for Au (b) and 530 nm for Ag (d)) are characterized by strong field confinement at the bottom of the particle, at the interface with the oxide layer. The effect can be observed most clearly in the Ag nanoparticle, as the resonances in the Au overlap with the interband transitions in the metal. This excitation of two resonances localized at different interfaces in the presence of high refractive index substrates has also been reported for Ag nanoparticles on Si substrates.[21] Finally, in Figure S2 (e) is the field plot

corresponding to the plasmon resonance localized at the bottom of the Al nanoparticle. In this material we have not observed a plasmon resonance localized at the top of the particle within the calculated spectral range, but nevertheless expect it to exist at shorter wavelengths.
